# Supplementary material for: Ftsh Sensitizes Methicillin-Resistant Staphylococcus aureus to β-Lactam Antibiotics by Degrading YpfP, a Lipoteichoic Acid Synthesis Enzyme
Source: Antibiotics (Basel). 2021 Oct 1;10(10):1198. doi: 10.3390/antibiotics10101198 (PMC8532640; doi:10.3390/antibiotics10101198)
Supplement: Supplementary file 1 [file antibiotics-10-01198-s001.zip › antibiotics-1370116-supplementary.pdf]

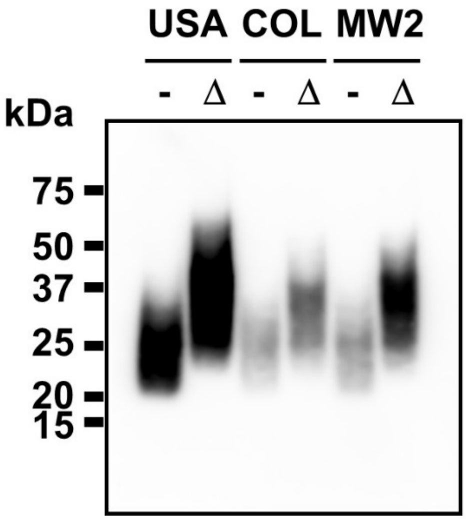

Fig. S1. The effect of the *ypfP*-deletion on the LTA expression in three different *S. aureus* strains. -, wild-type; *Δ*, a *ypfP*-transposon insertion mutant. USA, USA300.

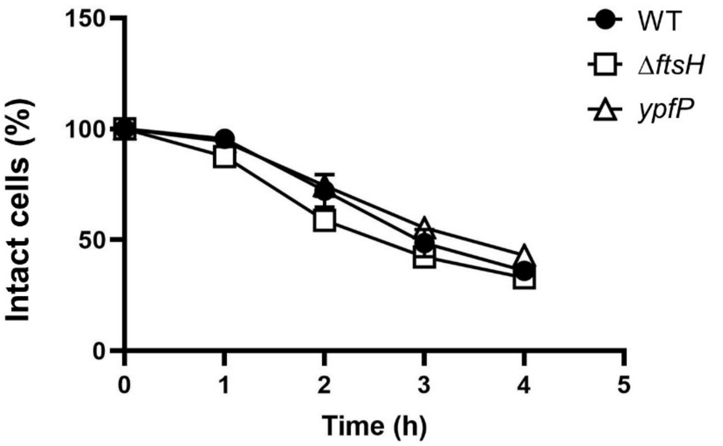

Fig. S2. The effect of the *ftsH* and the *ypfP* mutation on the autolysis activity of *S. aureus* USA300. WT, wild-type; Δ*ftsH*, *ftsH*-deletion mutant; *ypfP*, a *ypfP* transposon insertion mutant.

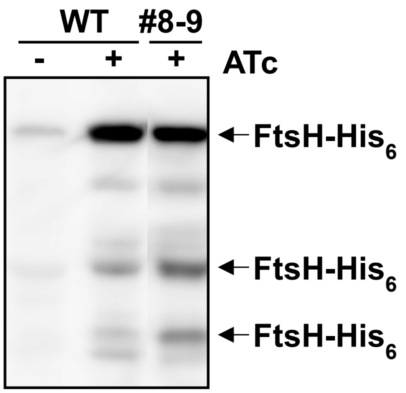

Fig. S3. FtsH expression in the wild-type and the suppressor mutant #8-9. All the strains contain the FtsH-expression plasmid pYJ-ftsH-His$_6$. FtsH expression was examined with an anti-His$_6$ antibody. ATc, anhydrotetracycline (100 ng/ml). Note that FtsH appears to be self-processed, as described previously {Akiyama, 1999 #6524}.

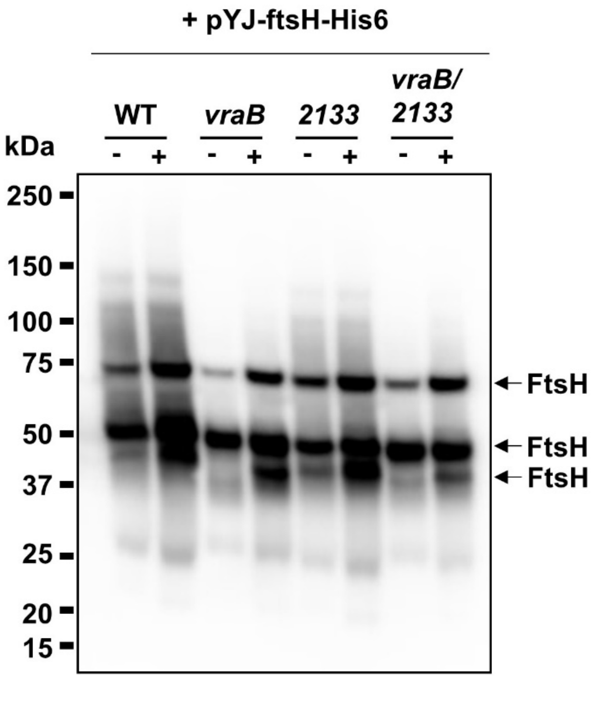

Fig. S4 FtsH expressions in the wild-type, *vraB*, *2133*, and the *vraB*/*2133* double mutant containing pYJ-ftsH-His6. FtsH expression was examined with an anti-FtsH antibody. The FtsH protein and its fragment species are indicated with arrows. -, no anhydrotetracycline; +, the addition of anhydrotetracycline. WT, USA300 wild-type; *vraB*, USA300::*vraB*(V144S); 2133, USA300::2133(V220L)

Table S1. Oxacillin MICs of FtsH-target gene mutants of USA300.

Mutated gene	Gene product	MIC (μg/ml)
-*		8
lrgB	**antiholin-like protein LrgB**	**4**
0310	hypothetical protein	8
0579	hypothetical protein	8
saeQ	SaeS regulatory protein SaeQ	8
ypfP	**diacylglycerol glucosyltransferase**	**0.5**
cydA	cytochrome D ubiquinol oxidase, subunit I	8
cyoE	protoheme IX farnesyltransferase	8
hemA	glutamyl-tRNA reductase	8
htrA	putative serine protease	8
mbtS	membrane-bound transcription factor	8
hslO	Hsp33-like chaperonin	8
hrtA	ATP-binding protein	8
hrtB	ABC transporter permease protein	8
2637	conserved hypothetical protein	8

* **-,** no mutation

Table S2. β-lactam resistance of *ypfP* mutants in three MRSA strain backgrounds.

**Strains**	**MIC (μg/ml)**		
	**Oxacillin**	**Cefotaxime**	**Cefazolin**
**USA300**	8	32	32
**USA300::*ypfP***	0.5	2	0.5
**COL**	256	128	32
**COL::*ypfP***	0.125	2	0.5
**MW2**	8	32	128
**MW2::*ypfP***	0.125	2	0.5

Table S3. The effect of the *ypfP*-deletion and the core suppressor mutations on the oxacillin MIC of the USA300.

Strains	MIC (µg/ml)
WT	8
ΔftsH	64
ΔypfP	0.5
ΔftsHΔypfP	0.5
WT (pYJ-*ftsH*-His_6)	0.5
#8-9*	32
#8-13*	32
#8-9C**	32
#8-9C::*ypfP*	0.125

* These strains contain pYJ-ftsH-His_6

** #8-9 without pYJ-ftsH-His_6

Table S4. Bacterial strains and plasmids used in this study

Strain/ plasmid	Relevant characteristic	Source
E. coli		
DH5α	Plasmid free, Lac-	New England Biolabs
S. aureus		
RN4220	Restriction deficient, prophage cured	[48]
USA300-0114	Clinical isolate	NARSA*
COL	Clinical isolate	[33]
MW2	Clinical isolate	[34]
USA300-P23	USA300-0114 without plasmid 2 and 3	[49]
USA300*ΔftsH*	*ftsH*-deletion mutant of USA300-P23	[21]
USA300::*mecA*	Transposon insertion in *mecA* in NE1868 transduced into USA300-P23	This study
USA300*ΔftsH::mecA*	Transposon insertion in *mecA* in NE1868 transduced into USA300*ΔftsH*	This study
USA300::*ypfP*	Transposon insertion in *ypfP* in NE1663 was transduced into USA300-P23	This study

USA300::*lrgB*	Transposon insertion in *lrgB* in NE1726 transduced into USA300-P23	This study
USA300::0310	Transposon insertion in SAUSA300_0310 in NE1254 transduced into USA300-P23	This study
USA300::0579	Transposon insertion in SAUSA300_0579 in NE219 transduced into USA300-P23	This study
USA300::*saeQ*	Transposon insertion in *saeQ* in NE1549 transduced into USA300-P23	This study
USA300::*cydA*	Transposon insertion in *cydA* in NE117 transduced into USA300-P23	This study
USA300::*cyoE*	Transposon insertion in *cyoE* in NE1434 transduced into USA300-P23	This study
USA300::*hemA*	Transposon insertion in *hemA* in NE1742 transduced into USA300-P23	This study
USA300::*htrA*	Transposon insertion in *htrA* in NE1141 transduced into USA300-P23	This study
USA300::*mbtS*	Transposon insertion in *mbtS* in NE132 transduced into USA300-P23	This study
USA300::*hslO*	Transposon insertion in *hslO* in NE1128 transduced into USA300-P23	This study

USA300::*hrtA*	Transposon insertion in *hrtA* in NE1489 transduced into USA300-P23	This study
USA300::*hrtB*	Transposon insertion in *hrtB* in ΦNΞ-8752 transduced into USA300-P23	This study
USA300::2637	Transposon insertion in SAUSA300_2637 in NE1164 transduced into USA300-P23	This study
USA300*ΔftsH::ypfP*	Transposon insertion in *ypfP* in NE1663 transduced into USA300*ΔftsH*	This study
COL::*ypfP*	Transposon insertion in *ypfP* in NE1663 was transduced into COL	This study
MW2::*ypfP*	Transposon insertion in *ypfP* in NE1663 was transduced into MW2	This study
#8-9	A suppressor mutant of USA300*ΔftsH* (pYJ-*ftsH*-His_6) whose oxacillin MIC is not affected by FtsH overexpression	This study
#8-13	Another suppressor mutant of USA300*ΔftsH* (pYJ-*ftsH*-His_6) whose oxacillin MIC is not affected by FtsH overexpression	This study
#8-9C	#8-9 without pYJ-*ftsH*-His_6	This study
USA300::*vraB*(V144S)	The *vraB* (V144S) mutation was introduced into USA300-P23	This study

USA300::2133(V220L)	The SAUSA300_2133 (V220L) mutation was introduced into USA300-P23	This study
USA300:: *vraB*(V144S) /2133(V220L)	The *vraB* (V144S) and the SAUSA300_2133 (V220L) mutation were introduced into USA300-P23	This study
#8-9C::*ypfP*	Transposon insertion in *ypfP* in NE1663 was transduced into #8-9C	This study
**Plasmids**		
pYJ335	An *E. coli-S. aureus* shuttle vector with anhydrotetracycline-inducible promoter	[50]
pYJ-ftsH-His_6	pYJ335 containing the *ftsH* gene with His-tag sequence at the C-terminus	[20]
pKOR1	An allelic replacement plasmid for *S. aureus*	[40]
pKOR1-vraB	pKOR1 carrying a 2-kb fragment with the *vraB* (V144S) mutation	This study
pKOR1-2133	pKOR1 carrying a 2-kb fragment with the SAUSA300_2133 (V220L) mutation	This study

* Network on Antimicrobial Resistance in *Staphylococcus aureus.*

Table S5. Oligonucleotides used in this study

Name	Sequence (5′ > 3′)	Target
P3764	TAAAATAAGCTTGATATCGATGGGAAGTAGGAGGAAATG	*ftsH*
P3765	TGACATTAGAAAACCGACTGCGTACTTCCAATCCAATGC	*ftsH*
P3766	AGCATTGGATTGGAAGTACGCAGTCGGTTTTCTAATGTCAC	pYJ335
P3767	TCATTTCCTCCTACTTCCCATCGATATCAAGC	pYJ335
P4043	CCAAGCCGGAGCTGGC	*vraB*
P4044	GGAATATGTGATTTTAGACTTTCATCAGTGTTG	*vraB*
P4045	GGCAATGCCTCAGTACGCG	2133
P4046	ATTGCCAGGACGTTCGACGG	2133
P4203	CTGCTAGCTAGCTAGAGATGCAGTGGAGATAAGCTGCATTCTTC	*vraB*
P4204	AACCGGTACCAATGGATCATTAAACTTCATCTCTACTTCATCGCC	*vraB*
P4205	CTGCTAGCTAGCTAGAGATGGATGTGTTTGAATAATGGCAATGACAG	2133
P4206	AACCGGTACCAATGGATGCCCGTGGCATTCATCCG	2133
P4246	ACGTTGAGCCTCGGAACCGG	pKOR1
P4248	GTTAGTTTACAACAATGAACATACAATCCAATC	*vraB*
P4249	GTATCACATAACTCTTGAAAACGATTACAAAATCG	2133
